# Barriers and Facilitators to Implementing Pressure Injury Guidelines for Nutrition Assessment and Alternating Pressure Air Mattress Allocation: A Qualitative Study

**DOI:** 10.1111/jan.16820

**Published:** 2025-02-12

**Authors:** Ching Shan Wan, Mika Musgrave‐Takeda, Brigid M. Gillespie, Georgia Tobiano, Elizabeth Mcinnes

**Affiliations:** ^1^ Respiratory Research@Alfred, School of Translational Medicine Monash University Melbourne Victoria Australia; ^2^ Nursing Research Institute, St Vincent's Health Network Sydney, St Vincent's Hospital Melbourne & Australian Catholic University Fitzroy Victoria Australia; ^3^ National Health and Medical Research Council Centre of Research Excellence in Wiser Wound Care Griffith University Gold Coast Queensland Australia; ^4^ School of Nursing, Midwifery and Paramedicine Australian Catholic University Melbourne Victoria Australia; ^5^ Gold Coast University Hospital Gold Coast Health Nursing and Midwifery Education and Research Unit Gold Coast Queensland Australia

**Keywords:** evidence‐based practice, guideline adherence, implementation science, interdisciplinary research, malnutrition, pressure ulcer, qualitative research, support surface

## Abstract

**Aims:**

To investigate clinicians' views on barriers and facilitators to implementing pressure injury prevention guideline recommendations for nutrition assessment and treatment, and de‐implementing inappropriate alternating pressure air mattress allocation.

**Design:**

A qualitative descriptive study adhering to the COnsolidated criteria for REporting Qualitative research (COREQ) guidelines.

**Methods:**

We conducted face‐to‐face or videoconference focus groups and semi‐structured individual interviews with clinicians recruited from a metropolitan tertiary hospital. Participants were purposively sampled according to their years of clinical practice. Interview transcripts were thematically analysed inductively to derive barriers and facilitators to guideline uptake. These were then mapped to the Theoretical Domains Framework and behaviour change techniques to inform an evidence‐based implementation intervention development to improve guideline uptake.

**Results:**

Thirteen nurses, four occupational therapists and three dietitians were interviewed. Six themes illustrate three guideline‐specific barriers and three common facilitators influencing nutrition‐ and mattress‐related guideline uptake. The three barriers were: (1) nurses devalue the use of validated tools in nutrition screening; (2) nurses prioritise vital‐sign‐related nursing duties over feeding assistance according to clinical urgency; and (3) nurses consider air mattresses a preventative strategy irrespective of patient PI risks. Facilitators to improve guideline uptake were: (1) nurse‐led interdisciplinary collaboration, (2) carer involvement and (3) easily accessible updated guidelines. Different Theoretical Domains Framework domains and behaviour change techniques were mapped to the identified nutrition‐ and mattress‐related barriers.

**Conclusion:**

The findings highlight three key nurses' attitudinal barriers to nutrition‐ and mattress‐related guideline uptake, which inform the development of theory‐ and end‐user‐informed implementation interventions in pressure injury prevention.

**Implications:**

An implementation strategical plan that addresses attitudinal barriers to improving guideline uptake for nutrition assessment and treatment and reducing air mattress overprescription appears critical in developing an intervention to enhance value‐based practice, which will need to be evaluated in future trials.

**Patient or Public Contribution:**

No Patient or Public Contribution.

**Summary**

- What does this paper contribute to the wider global clinical community?
  ○ Addressing nurses' attitudinal barriers about devaluing the use of structured nutritional screening techniques to support nursing clinical judgement in dietitian referrals is important for improving the pressure injury guideline uptake related to nutritional assessment.
  ○ Nurses' and junior occupational therapists' acceptance of alternating pressure air mattress overprescription in patients at low risk of pressure injuries needs to be addressed to reduce unnecessary resource waste and improve value‐based pressure injury prevention.
  ○ Nurses, dietitians and occupational therapists believe enhancing the accessibility of updated clinical guidelines and fostering nurse‐allied health interdisciplinary collaboration improves the uptake of pressure injury guidelines, which warrants future research on the effectiveness of these facilitators in an intervention bundle.

## 1 Introduction

Patient safety is fundamental to delivering quality health services (Australian Commission on Safety and Quality in Health Care 2019). One common hospital patient safety indicator is pressure injuries (PIs), defined as localised skin and/or underlying tissue damage resulting from pressure with/without shear (Pan Pacific Pressure Injury Alliance 2019). PIs are preventable if PI clinical guidelines are followed (Nghiem et al. 2022). However, low adherence to pressure injury prevention clinical guidelines is common even when a well‐established PI prevention system has been implemented in complex, resource‐limited and priority‐driven acute care hospital settings (Lovegrove et al. 2021b). Factors contributing to guideline uptake include practical difficulties such as limited staff capacity, inadequate knowledge and skills, and equipment availability in guideline implementation and suboptimal nurse‐led multidisciplinary care (Wan et al. 2023).

Currently, PI affects 12.8% of adult patients admitted to hospitals worldwide, causing physical patient harm, reducing patient quality of life and increasing the financial burden on hospitals and patients (Li et al. 2020). In 2020, the estimated total cost of PI in the Australian public hospital system was approximately $9.11 billion (Nghiem et al. 2022), emphasising the significance of PI prevention in reducing unnecessary healthcare burden and improving patient health outcomes and safety. The most recent systematic review that investigated factors influencing the efficacy of the PI prevention intervention bundle has highlighted the need for a theory‐and evidence‐informed implementation strategical plan as part of the intervention bundle to address clinicians' perceived barriers to the PI prevention guideline uptake (Chaboyer et al. 2024). Yet, it has been found that existing PI prevention intervention bundles targeting acute care hospital settings were of limited rigour, not end‐user‐informed and theory‐driven in addressing clinicians' perceived barriers to the PI prevention guideline uptake (Lovegrove et al. 2021a).

Barriers to the guideline uptake identified by clinicians are PI prevention strategy‐specific (Wan et al. 2023). Given the challenges in facilitating interdisciplinary collaboration in complex acute care settings (Al‐Dosari et al. 2022), it is not surprising to observe the low adherence to guidelines on PI prevention strategies that require interdisciplinary collaboration, including nutrition assessment and treatment (Citty et al. 2019) and alternating pressure air mattresses (APAM) prescription (Bååth et al. 2024). Currently, despite malnutrition being an independent risk factor of PI (Chen et al. 2023), not all patients at high risk of PI are screened for malnutrition, and not all patients at high risk of both PI and malnutrition receive timely nutritional treatment (Citty et al. 2019). Contemporaneously, the overuse of APAM in replacing frequent repositioning is also observed in acute care settings (Bååth et al. 2024), which is considered low‐value care because it is not aligned with evidence‐based guidelines (also described as evidence‐practice gaps), incurs unnecessary healthcare costs and wastes limited health service resources (Kim et al. 2021).

Choosing Wisely De‐implementation Framework is a comprehensive approach to systematically reduce low‐value care that builds upon previous de‐implementation research (Grimshaw et al. 2020). Recommended by the Choosing Wisely De‐implementation Framework, identifying the drivers of these evidence‐practice gaps by exploring clinicians' views on barriers and facilitators to evidence‐based PI prevention care is the first critical step for designing a targeted implementation intervention bundle to improve the uptake of evidence in clinical practice. In addition, early engagement of multidisciplinary end‐users for clinical practice change could promote the uptake of guidelines for ‘should do’ and ‘do not do’ practices (Grimshaw et al. 2020). Therefore, this study was designed as part of the program of research to inform the development of a theory‐ and end‐user‐informed intervention to address the identified evidence‐practice gaps in PI prevention in acute care settings.

## 2 Aim

The aim of this study was to explore hospital bedside nurses', dietitians' and occupational therapists' views on barriers and facilitators to implementing PI prevention guideline recommendations for nutrition screening and treatment and de‐implementing inappropriate APAM allocation. To inform the development of an intervention to improve the PI prevention guideline uptake, the barriers and facilitators identified in this study were then mapped to the Theoretical Domains Framework and behaviour change techniques for the transparency of reporting barriers and facilitators. The study was conducted and reported using the consolidated criteria for reporting qualitative research (COREQ) reporting guidelines (Tong et al. 2007).

## 3 Methods

### 3.1 Design

A qualitative descriptive study design using both semi‐structured focus groups and individual interviews was used to elicit rich and in‐depth data on clinicians' views on barriers and facilitators to implementing PI prevention guideline recommendations for nutrition screening and treatment and de‐implementing APAM overprescription (Kim et al. 2017).

### 3.2 Theoretical Frameworks Used

Underpinned by the Choosing Wisely De‐implementation Framework (Grimshaw et al. 2020), we used an end‐user‐targeted and evidence‐informed approach to firstly explore the barriers and facilitators to implementing nutrition‐ and APAM‐related PI prevention guidelines from clinicians' perspectives, followed by using implementation science frameworks or models to systematically develop more scalable and sustainable interventions (Milat et al. 2015).

The behaviour change wheel (BCW), which is a synthesis of 19 existing behaviour change frameworks identified from systematic reviews (Michie et al. 2011), provides a systematic and transparent approach for designing a theory‐based evidence‐informed intervention (Lavallée et al. 2019; Michie et al. 2014). The Capability, Opportunity and Motivation—Behaviour model forms the core of the behaviour change wheel and links the commonly used Theoretical Domains Framework and behaviour change techniques in clinical practice change intervention development (Cane et al. 2012; Cane et al. 2015; Michie et al. 2011). The Theoretical Domains Framework enables the identification of behavioural determinants that influence PI prevention guideline uptake (Lavallée et al. 2018). The behaviour change techniques allocation is based on the Capability, Opportunity and Motivation—Behaviour model and the Theoretical Domains Framework, and allows the identification of active components of evidence‐informed strategies that target clinicians' perceived barriers to implementing PI prevention guidelines (Bérubé et al. 2015). The Theoretical Domains Framework and behaviour change techniques have only been used in PI intervention development in community settings (Lavallée et al. 2018; Taylor et al. 2021).

### 3.3 Setting and Participants

To understand clinicians' views on barriers and facilitators to implement PI prevention guidelines for nutrition screening and treatment and APAM allocation in acute care settings, clinicians involved in nutrition and APAM decision‐making in a metropolitan Australian tertiary teaching hospital were selected, including bedside nurses, dietitians and occupational therapists. This study commenced on 1 April 2022 after COVID‐19 restrictions in hospitals were lifted. Purposive sampling was used to recruit bedside nurses, dietitians and occupational therapists who were working in selected surgical, medical, subacute and geriatric rehabilitation wards. Clinicians with more than 1 year of post‐qualification experience and who regularly provided care for patients with or at high risk of PI were eligible. This sampling method enables the collection of adequate (richness and complexity) data from broad key health disciplines to address the research question. Student clinicians and clinicians who practised on wards other than these four selected wards were excluded.

The Nurse Unit Managers of the selected four wards and the allied health managers were asked to nominate potential participants who met the eligibility criteria. Potential participants were approached via invitation emails with an attached participant information sheet and consent form requesting consideration of involvement in this study. All nominated potential participants except one dietitian had expressed their interest in participating, and written informed consent was obtained. The final sample size was determined based on whether a maximum variation in clinical experience and role and the type of wards practised were recruited to ensure heterogeneity regarding clinical characteristics. Once it was determined that no new dimensions, nuances or insights of issues were described by participants in the focus groups and interviews (meaning data sufficiency), recruitment was ceased (Braun and Clarke, 2021c).

### 3.4 Data Collection

The participants were first asked to complete a demographic questionnaire before focus groups or individual interviews to collect information on their current clinical position, the highest level of education, speciality qualification, years of practice in the profession since graduation and current position and the frequency of seeing patients with or at high risk of PI.

The semi‐structured focus group and interview guides were developed to explore participants' views on factors influencing the uptake of international guideline recommendations (shown in Table 1) for nutrition screening and treatment and appropriate allocation of APAMs. Separate questions were designed for nutrition‐ and APAM‐related PI prevention practices (detailed in Table 2). Since team nursing was practised in hospital PI prevention care provision, semi‐structured focus groups were conducted with nurses. As hospital patients were assigned to individual allied health in PI prevention care provision, semi‐structured individual interviews were conducted with dietitians and occupational therapists. Focus groups and interviews were conducted either face‐to‐face or videoconference at a mutually convenient time and place. Each interview was conducted by the same experienced qualitative research dietitian (CSW) who was not a hospital employee to ensure consistency. The focus groups and interviews were audio‐recorded, transcribed verbatim by a professional transcription company and proofread by a research assistant (MM). Transcripts were de‐identified, and names were removed and replaced with a unique code to maintain anonymity.

**TABLE 1:** Nutrition‐ and alternating pressure air mattress‐related pressure injury guideline recommendations of interest.

Guidelines (Pan Pacific Pressure Injury Alliance 2019)	Evidence‐practice gaps
**(A) Nutrition‐related guideline recommendations**	
Conduct nutritional screening for individuals at risk of a pressure injury.	Not all patients at risk of pressure injuries have completed nutritional screening (Roberts et al. 2015).
2 Conduct a comprehensive nutrition assessment for adults at risk of a pressure injury who are screened to be at risk of malnutrition.	Not all patients at risk of pressure injuries and malnutrition are referred to see a dietitian for comprehensive nutrition assessment (Roberts et al. 2015).
3 Develop and implement an individualised nutrition care plan for individuals at risk of a pressure injury who are malnourished or at risk of malnutrition.	Not all patients at risk of pressure injuries and malnutrition are provided with an individualised nutrition care plan (Fulbrook et al. 2024).
**(B) Alternating pressure air mattress‐related guideline recommendations**	
Use a high specification reactive single layer foam mattress or overlay in preference to a foam mattress without high specification qualities for individuals at risk of developing pressure injuries.	Patients at low risk of pressure injuries are inappropriately allocated to expensive alternating pressure air mattresses (Latimer et al. 2014).
2 Assess the relative benefits of using an alternating pressure air mattress or overlay for individuals at risk of pressure injuries.	Alternating pressure air mattresses allocation is not appropriate in clinical practice (Rosenberg 2022).

**TABLE 2:** Semi‐structured interview and focus group guide.

Questions	Prompts
**(A) Nutrition‐related**	
(1) Could you tell me about the dietitian referral process for patients at high risk of PIs?	PI screening, malnutrition screening, risk status reassessment
(2) Do all referred patients at high risk of PI and malnutrition receive a comprehensive nutrition assessment and nutrition care plan?	Percentage of patients being seen by dietitians, how long would a patient need to wait to be seen by dietitians
(3) What is the pathway/ follow‐up if a malnourished patient at high risk of PI is provided with a nutrition care plan?	Meal/ oral nutrition support ordering, implementing, monitoring and reviewing nutrition care plan
(4) Who is involved in risk assessments, referring dietitians, implementing and following‐up nutrition care plans?	Communication between multidisciplinary team, dietary assistance, completing food charts, nutrition status reassessment
(5) What is your understanding about nutrition‐related PI prevention guidelines/ hospital policies?	Benefits and drawbacks of following guidelines from patient, clinician and hospital perspectives
(6) Whose responsibility do you think it is to implement these guidelines?	Malnutrition screening, nutrition assessment, implementing care plan
(7) How easy/difficult is it to implement these guidelines?	Barriers and facilitators, communication within and between health disciplines, patient involvement
(8) How can we overcome the barriers?	Strategies to address individual, group and organisational barriers
**(B) Mattresses‐related**	
(1) Do all patients who are at high risk of PI have allocated a specialised mattress? How about patients who are at low risk of PI?	Percentage of patients at high and low risk of PI being allocated a specialised mattress, percentage of patients being seen by occupational therapists
(2) What is your views on whether specialised mattresses are used appropriately or over‐ordered?	PI risk assessment and reassessment, occupational therapist referrals
(3) What are the criteria for allocating a patient to a specialised mattress?	Hospital/ward policy, level of PI risk, patient request, clinical judgement
(4) Who is involved in making decisions about the use of specialised mattresses and mattresses ordering/allocation process?	Mattresses ordering process, communication within and between health disciplines
(5) What is your understanding about the most recent evidence/ guideline recommendations for the use of mattresses in PI prevention?	Benefits and drawbacks of following guidelines from patient, clinician and hospital perspectives
(6) Whose responsibility do you think it is to implement these guidelines?	Mattress ordering, multidisciplinary teamwork and shared decision‐making
(7) How easy/difficult is it to implement these guidelines?	Barriers and facilitators, communication within and between health disciplines, patient involvement
(8) How can we overcome the barriers?	Strategies to address individual, group and organisational barriers

Abbreviation: PI, Pressure injury.

### 3.5 Data Analysis

Demographic information was summarised and presented as descriptive data (counts, percentages). Qualitative data were first analysed inductively using the approach described by Braun and Clarke (2021a). Inductive reflexive thematic analysis was used to identify the barriers and facilitators described by participants through an interpretative lens. It involved six steps, which were (1) data familiarisation of the data, (2) data‐driven initial codes generation by aggregating data with similar meanings, (3) initial themes generation through sorting codes with similar focus and scope in each nutrition‐ and APAM‐related data, (4) reviewing and combining themes that were similar between nutrition‐ and APAM‐related data, (5) renaming themes to capture the essence of the analysis and 6) renaming codes that capture unique meaning to become subthemes under each theme (Braun and Clarke 2021a; Campbell et al. 2021). Data analysis was also conducted separately for each health discipline due to their different perspectives.

The second stage of data analysis involved deductively mapping the subthemes generated in the first stage to the Theoretical Domains Framework. These subthemes were renamed as barriers or facilitators to reflect their relevancy with the most appropriate constructs under the relevant Theoretical Domains Framework domains (Cane et al. 2012). Barriers and facilitators were categorised into the relevant Capability, Opportunity and Motivation—Behaviour system of the behaviour change wheel according to the Theoretical Domains Framework allocation (Michie et al. 2011). Barriers and facilitators to improving the PI prevention guideline uptake were then allocated to the most appropriate behaviour change techniques according to the matrix described in the behaviour change wheel that links the Theoretical Domains Framework and Capability, Opportunity and Motivation—Behaviour with behaviour change techniques (Cane et al. 2015; Michie et al. 2014). The affordability, practicability, effectiveness and cost‐effectiveness, acceptability, side‐effects and safety, equity criteria described in the behaviour change wheel were used as a guide to consider six aspects when selecting behaviour change techniques for appropriate intervention options, content and implementation options (Jenkins et al. 2018; Michie et al. 2014).

All data were thematically analysed by the experienced qualitative research dietitian who conducted the focus groups and interviews (CSW), followed by an independent analysis of half of the randomly selected transcripts by a second coder, research nurse assistant (MM), to check for agreement in generated themes to achieve credibility and rigour. After the thematic analysis, CSW, who had experience in Theoretical Domains Framework and behaviour change techniques mapping, conducted the Theoretical Domains Framework and behaviour change techniques allocation, followed by discussion and cross‐checking with a senior researcher with implementation science expertise (EM) to reach an agreement about Theoretical Domains Framework mapping. The data were managed in NVivo 12 (QSR International Pty Ltd 2018). CSW led the comparative data analysis, with close conferral with the research team to provide investigator triangulation and achieve agreements on the final findings.

### 3.6 Ethical Considerations

This study was given approval by the St Vincent's Hospital Melbourne Human Research Ethics Committee (HREC) (Ref: LRR 316/21) and the Australian Catholic University HREC (Ref: 2022‐2541R) on 22 February 2022.

### 3.7 Rigour

No relationship was established with participants before the study commencement to ensure the auditability of the data collection process. Participants recognised that the researcher who conducted the focus groups and interviews was not working at the hospital and did not know about the researcher's clinical background before and during data collection. This enhanced the trustworthiness of data collection by promoting participants' sincerity in unbiasedly describing current clinical practices. Discussing initial themes with the research assistant (MM) with a nursing background in the data analysis process helped the primary researcher (CSW) to self‐reflect on how personal propositions and assumptions (including dietetics background and familiarity of existing literature on barriers to the PI prevention guideline uptake) influenced the interpretation of data. This self‐reflexive approach in theme generation enhanced the credibility of data‐driven analysis (Braun and Clarke 2021b). To strengthen the credibility of behaviour change techniques mapping, the primary researcher's involvement in the hospital's clinical risk committees in the data analysis stage assisted the researcher in better considering the practicability and acceptability aspects when mapping barriers to relevant behaviour change techniques.

## 4 Findings

Thirteen bedside nurses, three dietitians and four occupational therapists were interviewed. Each focus group and interview took around an hour. Table 3 shows the characteristics of the participants stratified by the health discipline. Almost all participants were female. Most participants (95%) had completed their Bachelor's degree. Most participants (60%) had practised in their health profession for at least 7 years and in their current position for 4 years or more. Most participants (70%) saw patients at high risk of or having PI at least once a day.

**TABLE 3:** Participant demographics stratified by health disciplines.

Demographics	Bedside nurses (*n* = 13)	Occupational therapists (*n* = 4)	Dietitians (*n* = 3)
Female, *n* (%)	13 (100)	4 (100)	2 (66.7)
Clinical position, *n* (%)	RN; 9 (69)	Grade 1; 2 (50)	Grade 2; 2 (67)
	CNS; 3 (23)	Grade 3; 2 (50)	Grade 3; 1 (33)
	EN; 1 (8)		
Full‐time, *n* (%)	3 (23)	4 (100)	2 (67)
Level of education, *n* (%)			
Certificate III	1 (8)		
Bachelor	10 (77)		1 (33)
Postgraduate	2 (15)	4 (100)	2 (67)
Speciality qualification, *n* (%)	3 (23)	1 (25)	1 (33)
Years of practice in profession, *n* (%)			
1–3 years	2 (15)	2 (50)	
4–6 years	3 (23)		1 (33)
7–9 years	6 (46)	2 (50)	
≥ 10 years	2 (15)		2 (67)
Years of practice in current position, *n* (%)			
< 1 year		3 (75)	1 (33)
1–3 years	3 (23)	1 (25)	
4–6 years	5 (38)		
7–9 years	3 (23)		
≥ 10 years	2 (15)		2 (67)
Frequency of seeing patients at risk of or have pressure injuries, *n* (%)			
≥ once a day	7 (53)	4 (100)	3 (100)
≥ once a week	6 (46)		

Abbreviations: CNS, Clinical Nurse Specialist; EN, Enrolled nurse; RN, Registered nurse.

Six key themes with several subthemes generated under each key theme were identified. Themes 1 and 2 described the barriers specific to implementing PI prevention guidelines related to nutritional screening and treatment. Theme 3 described the barriers related to appropriate APAM allocation. The remaining three themes were related to (1) the perceived importance of a nurse‐led multidisciplinary model, (2) carer involvement and (3) accessible PI prevention guidelines in facilitating the PI prevention guideline uptake. Tables 4, 5, 6 show the subthemes supported by illustrative quotes described as barriers and facilitators to implementing nutrition‐ and APAM‐related guideline recommendations under each theme identified from nurses', dietitians' and occupational therapists' transcripts, respectively. Table S1 presents barriers and facilitators identified in each theme stratified by health disciplines.

**TABLE 4:** Bedside nurses' views on barriers and facilitators to preventing pressure injuries in relation to nutrition and mattresses mapped to the capacity, opportunity, motivation—behaviour model and Theoretical Domains Framework domains.

Capability, opportunity and motivation–behaviour	Theoretical Domains Framework (constructs)	Subtheme—Barrier/facilitator	Exemplar quote
**Theme 1: Devaluing of the use of pressure injury and malnutrition risk assessment tools**			
Motivation – Reflective	Goals (Goal priority)	Prioritise vital sign‐related nursing tasks over risk assessment completion—Barrier	‘Time can sometimes be a big issue. We are very busy up here, and unfortunately, sometimes the risk assessment might not be completed properly and may be missed… The risk screening might not be a priority for the patient. The main things will be ensuring they're vitally stable and giving them medications on time. So things get pushed back’. (NFG001)
Capability – Psychological	Memory, Attention and Decision Processes (Decision making)	Only use risk assessment tools as prompts when nurses feel they are unable to make a clinical judgement to decide if referral to allied health is needed—Barrier	‘We do nutrition referral when the patient has decreased appetite, not enough food intake, is very frail and bony, has renal issues… chronic wounds… dramatic weight loss as part of the reason for admission… patients who have seen by speech pathology… previously on PEG tube… Or patient says “I have lost weight recently.”’ (NFG001)
Capability –Psychological	Memory, Attention and Decision Processes (Attention control)	Do not consider malnutrition risk when assessing pressure injury risk because separate assessment tools are used—Barrier	‘I don't think we do it [pressure injury risk assessment] based on nutrition. We're doing it based on their skin integrity, mobility, age, weight and those types of things… I've always looked at someone and then you're malnourished. Let's refer you to the dietitian. I know malnutrition can make a call as a contributing factor, but I don't put that down to pressure injury risk’. (NFG002)
**Theme 2: Inadequately integrate nutrition care plan in medical treatment**			
Motivation – Reflective	Goals (goal priority)	Prioritise medical intervention over patient mealtime—Barrier	‘Sometimes they[patients] have appointments at lunchtime, and then they sometimes would miss out. But when they come back, we always offer. They have sandwiches, but they do miss meals with appointments’. (NFG004)
Opportunity – Physical	Environmental Context and Resources (Environmental stressors)	Incomplete food charts due to other nursing workload priorities and hence food intake monitoring is suboptimal—Barrier	‘It can be hard if you have six patients and need to feed a patient… You have to get everyone else set up and ensure you're okay before you can attend to the person who requires assistance… You cannot document the food chart if you're not there to check the tray before it gets taken’. (NFG004)
**Theme 3: Overuse of alternating pressure air mattresses**			
Motivation – Reflective	Intentions (Stability of intentions)	Alternating pressure air mattresses overuse is safer for patients than underuse to prevent pressure injuries—Barrier	‘Sometimes patients who are low risk were put on an air mattress… If we know that they will be full nursing care, we assume that they will also be high pressure injury risk… If they're stroke patients, we put them on an air mattress straightaway as well… Or if they're coming from a nursing home or a subacute, they kind of automatically go onto an air mattress… But we're pretty quick at getting air mattresses’. (SFG001)
Motivation – Reflective	Beliefs about Consequences (Outcome expectancies)	Some nurses prefer alternating pressure air mattresses to be the standard hospital mattress because most patients are at risk of pressure injuries—Barrier	‘Changing beds is just this huge process, as opposed to if we just owned our air mattresses it wouldn't be such a big deal with switching over… Not having to do so many orders, changeovers, and bed moves’. (SFG002)
**Theme 4: Nurses coordinating multidisciplinary care**			
Motivation – Reflective	Social/Professional Role and Identity (Professional role)	Importance of nurses' coordinating role in providing regular and reciprocal interactions between the multidisciplinary team in pressure injury prevention—Facilitator	‘I think both nurses and occupational therapists are involved in air mattresses. Generally, the nurses will organise the air mattress if the patient is at high risk. The occupational therapist will suggest whether the patient can move in the bed. If they can move in their bed, they don't need an air mattress’. (SFG004)
			‘Lots of coordinating work… Nurses are advocates for patients because dietitians are not around all the time… when the meal comes, we can set them up and help them, monitor what they like and dislike and relay that back to the dietitians’. (NFG004)
Motivation – Reflective	Social/Professional Role and Identity (Professional boundaries)	Lack of expertise to be solely responsible for making decisions regarding mattress allocation—Barrier	‘We are supposed to be experts in every allied health discipline. So, I think it is good that we could get education from occupational therapists on different mattresses and cushions… All nurses should be educated on how to prevent pressure injuries in terms of equipment’. (SFG002)
Motivation – Reflective	Social/Professional Role and Identity (Organisational commitment)	Some dietitians and occupational therapists work independently with little multidisciplinary communication—Barrier	‘Every time I ask an occupational therapist to reassess a patient, or the seating is inaccurate, or whatever the problem is, they always say either it's not their patient, or they'll do it later, or they're short‐staffed. Still, they'll never do anything about it, which is frustrating for us… I sympathise with this, but we're all short‐staffed. We need to make it work, and we need to help each other’. (SFG002)
**Theme 5: Facilitating patient participation in their care**			
Opportunity – Physical	Environmental Context and Resources (Resources)	Encourage family involvement in feeding patients and providing communication assistance between the patient and nurses—Facilitator	‘The risk assessment forms wouldn't get filled out if you've got a non‐English speaking patient… or they are confused. Then you can try family… If they have a cultural preference, we ask the family members to bring their food from home, and then they enjoy… They are very helpful in feeding and translating what the patients want to do’. (NFG004)
Motivation – Reflective	Social/Professional Role and Identity (Professional boundaries)	Not confident in involving patients in the nutrition care plans and mattress allocation decision‐making due to nurses' lack of expertise in nutrition and mattress—Barrier	‘Once dietitians see them develop a plan, patients do have an input of different flavours, like and dislike’. (NFG002)
			‘Patient said, “I don't like this chair cushion. Not comfortable.” But I said, ‘That's the one that the occupational therapist set up.’…I think it is good for nurses to get educated about things… mattresses and cushions’. (SFG002)
Motivation – Reflective	Beliefs about Consequences (Characteristics of outcome expectancies)	Challenging to involve patients in mattress decision‐making because patients dislike alternating pressure air mattresses regardless of pressure injury risk—Barrier	‘They [patients] always complain they're uncomfortable. They don't like the air mattress… because it's too lumpy, too uncomfortable, or the machines are too loud and noisy… We can't give them an option because it's for their benefit, but when you tell someone something, and they know about it, they complain more about it’. (SFG001)
**Theme 6: Beliefs about the value of pressure injury prevention guidelines**			
Motivation – Reflective	Beliefs about Consequences (Characteristics of outcome expectancies)	Following guidelines helps deliver positive outcomes without increasing workload—Facilitator	‘I feel if I don't turn them or I don't put them on an air mattress, it makes me worry more… If we follow the guidelines, it will reduce the risk of patient injury’. (SFG001)
Motivation – Reflective	Goals (Action planning)	Audit and feedback mechanism in a multidisciplinary meeting improves pressure injury care—Facilitator	‘Sometimes, when something does happen, we have to have a more formal look at why something happened. We might have a huddle with bedside nurses, occupational therapists, dietitians and the nurse in charge… Get the history and try and work out together’. (NFG001)
Opportunity – Physical	Environmental Context and Resources (Resources)	Ward‐based clinical champions lead and coordinate multidisciplinary pressure injury prevention—Facilitator	‘Having a pressure injury champion in each element… or leaders to take charge… nurses can give feedback to that particular staff… expert person to reach out. And because they are the champions, they are leading these things’. (SFG002)
Capability – Physical	Skills (Skills development)	Ongoing pressure injury prevention education and training for bedside nurses—Facilitator	‘We do have quite a lot of new staff every 6 months. So, if we don't have ongoing education from clinical nurse specialists or educators, they probably don't know how to assess patients' pressure areas or skin assessments’. (SFG004)

**TABLE 5:** Dietitians' views on barriers and facilitators to preventing pressure injuries in relation to nutrition mapped to the capacity, opportunity, motivation—behaviour model and Theoretical Domains Framework domains.

Capability, opportunity and motivation—behaviour	Theoretical Domains Framework (constructs)	Subtheme—Barrier/facilitator	Exemplar quote
**Theme: Devaluing of the use of pressure injury and malnutrition risk assessment tools**			
Motivation – Reflective	Beliefs about Consequences (Characteristics of outcome expectancies)	Missed referrals for patients at high risk of malnutrition because Malnutrition Screening Tools are incomplete or completed improperly by nurses—Barrier	‘I would say most of the time it's attempted… When looking through the notes, I'll see that someone's maybe half completed it, or they've completed it, but not scored it, or they've completed it, but not actioned a referral… So if it looks like they might be at risk of a pressure injury and perhaps some nutrition‐related needs, which often they do, I would give myself a referral’. (D002)
Motivation – Reflective	Social/Professional Role and Identity (Professional role)	Nurses overlook the role of nutrition care in pressure injury prevention—Barrier	‘For pressure injury related referrals, we never receive a referral until they have a pressure injury. So it's very reactive. It's not proactive… In the hospital, everyone often thinks pressure injuries are the occupational therapist, physiotherapist, and nursing staff's role. Nutrition's often forgotten and a bit behind in proactively getting involved’. (D003)
**Theme: Inadequately integrate nutrition care plan in medical treatment**			
Opportunity – Physical	Environmental Context and Resources (Organisational culture)	Hospitals need to raise awareness of nutrition in pressure injury prevention among the multidisciplinary team—Facilitator	‘It might be just more creating awareness of pressure injury prevention… The other thing could be to have some sort of wound lecture second yearly or whatever, where you bring that into the forefront of people's minds… It certainly dropped off the radar the last few years due to the pandemic’. (D001)
Motivation – Reflective	Social/Professional Role and Identity (Professional identity)	Monitor and encourage dietary intake of patients who are at risk of malnutrition during mealtimes—Facilitator	‘Nurses are really good here to try to push food on them [patients] during their meal, getting them set up. That's why I am trying to be there for lunch, too. So even just to walk around and to help people… Particularly with patients who are delirious or have dementia, at least you can watch them eat and get an idea, which is always handy and monitoring how they're going’. (D003)
**Theme: Nurses coordinating multidisciplinary care**			
Motivation – Reflective	Social/Professional Role and Identity (Professional role)	Collaborative teamwork between nurses, allied health and food service staff is important for optimising nutrition care for pressure injury prevention—Facilitator	‘The first thing would be the dietitian, and alongside that would be the food services staff or dietitian assistants to put that into motion as supplements that meet patients' preferences are sent correctly… Nursing and allied health assistants were also involved and asked to feed and encourage patients with their meals as well… Food services staff push a tray, see what's going on, and say, ‘Why have you not eaten?’ So I think all of us are involved in day‐to‐day monitoring’. (D001)
**Theme: Facilitating patient participation in their care**			
Motivation – Reflective	Beliefs about Capabilities (Perceived competence)	Value patient or family involvement in food chart completion—Facilitator	‘Occasionally, I will encourage patients to complete food charts, especially if they are capable of doing that themselves. I'll get them to fill out their own food chart to alleviate that job from the nursing staff… Most patients would be able to quite accurately complete their food charts, especially if you explain to them the reasoning and the rationale behind… Obviously, they have to have the cognitive capacity to do it’. (D002)
**Theme: Beliefs about the value of pressure injury prevention guidelines**			
Motivation – Reflective	Beliefs about Consequences (Characteristics of outcome expectancies)	Following pressure injury prevention guidelines is essential despite the increased workload in conducting nutritional assessments—Facilitator	‘Optimising nutrition can give them that quality of life and reduce mortality… Those kinds of benefits would be considerable for patient care… We also reduce the number of patients that really need a lot of staff. So we have good systems in place, then we have a better means of managing more patients’. (D001)
Motivation – Reflective	Goals (Action planning)	Audit and feedback mechanism in a multidisciplinary meeting improves pressure injury care—Facilitator	‘I think a monthly audit of pressure injuries would bring the topic of it much more front and centre for the team and raise awareness of it… Those referrals will be generated when things are in front of people's minds. So I think that would be an enabler with the guidelines’. (D002)
Opportunity – Physical	Environmental Context and Resources (Resources)	Pressure injury prevention clinical champion in each ward monitors the appropriateness of dietitian referral—Facilitator	‘It probably needs a champion on each ward to actually make sure that all the nutritional screening are filled in… It just probably needs to have enough staff in consideration and more awareness… Even having a nursing champion being employed once a week around the hospital if you don't have one person for the ward, that might be worth the investment’. (D001)

**TABLE 6:** Occupational therapists' views on barriers and facilitators to preventing pressure injuries in relation to mattresses mapped to the capacity, opportunity, motivation—Behaviour model and Theoretical Domains Framework domains.

Capability, opportunity and motivation—behaviour	Theoretical Domains Framework (constructs)	Subtheme—Barrier/facilitator	Exemplar quote
**Theme: Overuse of alternating pressure air mattresses**			
Capability – Psychological	Knowledge (Knowledge)	Over‐ordering of alternating pressure air mattresses by nurses due to easy accessibility and lack of knowledge of the benefits and drawbacks of all mattress options among nurses and junior occupational therapists—Barrier	‘I think they [prescribing air mattresses] can sometimes be overdone. When a patient is quite well and cognitively intact, from an occupational therapist's point of view, I would like my patients to not rely on specialised equipment, if possible… Because of the air mattress availability, if a patient is going, nurses might change the name of that air mattress to the new patient… There's more follow‐up for us, and then there's more follow‐up for the nurses as well because they've got to rearrange things around’. (OT002)
Opportunity – Physical	Environmental Context and Resources (Resources)	Hospital guidelines direct nurses to use alternating pressure air mattresses in patients at high risk of pressure injuries regardless of patient independence—Barrier	‘More difficulty would be sourcing, depending on what type of equipment you're trying to source. Sometimes some of [special equipment] are a bit hard to get, but it would be more come with sourcing it rather than implementing those guidelines into practice’. (OT001)
			‘I would say our hospital guideline directs us more towards air alternating mattress’. (OT004)
Motivation – Reflective	Social/Professional Role and Identity (Professional role)	Nurses concern about bearing risks associated with downgrading mattresses—Barrier	‘If we take the air mattress off, their skin could deteriorate, and there is a risk involved… We want to prepare people as best as possible for that transition home… We often take more clinical risk in anticipation of that transition home, whereas the nursing staff feel responsible for the risk here and now, so they will be less likely to take the risk. So it's balancing what's in the patient's best interest and what's going to make the most sustainable discharge plan for a longer term” (OT003)
**Theme: Nurses coordinating multidisciplinary care**			
Motivation – Reflective	Social/Professional Role and Identity (Professional role)	Clarification of the role of the multidisciplinary team in relation to prescription and monitoring the use of mattresses—Facilitator	‘Regarding the pressure mattresses, mostly it's the nursing staff. But if the pressure injuries are staged a little bit higher, we might come in and put our specialised knowledge on various equipment types… We would step in where we feel the pressure care risks are a little bit too high, and they need an air mattress or whether they're ready to grade down’. (OT002)
Motivation – Reflective	Social/Professional Role and Identity (Professional identity)	Input from multidisciplinary team comprising nurses and occupational therapists to enable shared decision‐making in prescribing appropriate mattresses—Facilitator	‘It's definitely a multidisciplinary approach… It's really holistic. We look at many different things and put all the puzzle pieces together… Communicating, ensuring the nurses are aware and then selecting an appropriate surface… Nurses are the first point of contact within the first eight hours of completing a full skin review’. (OT001)
Opportunity – Physical	Environmental Context and Resources (Person × environment interaction)	Suboptimal multidisciplinary communication and intradisciplinary handover of patient's pressure injury prevention plan—Barrier	‘But I would say probably equally, we would have a percentage where we are missing that risk and missing out on that referral opportunity where perhaps the patient is at a higher risk, and we may not get a referral until a pressure injury has been developed… One of the biggest challenges is to hand over effectively and ensure everything's in place in the acute ward. We don't always get a notice that somebody's going to transfer to subacute, and we may not always get clear directions as to which subacute ward they're going to’. (OT004)
**Theme: Facilitating patient participation in their care**			
Motivation – Reflective	Beliefs about Capabilities (Perceived competence)	Encourage shared decision‐making in allocating appropriate mattresses by involving patients and/or carers early—Facilitator	‘Many patients report that it is uncomfortable to be on an air mattress and cold and noisy…We've just got to try and implement some strategies to get them to start to weight shift on their own to prevent those pressure care issues… I will provide them with handouts, and verbal education, practising that on the ward, seeing if the family can communicate that to the patient and see if the patient is receptive to their communication… Listen to what the patient wants and needs.’ (OT002)
**Theme: Beliefs about the value of pressure injury prevention guidelines**			
Motivation – Reflective	Beliefs about Consequences (Characteristics of outcome expectancies)	Prefer following guidelines because the benefits of following guidelines outweigh the drawbacks—Facilitator	‘It can be tricky balancing workload… Hiring these different cushions or specialised mattresses can be costly. But if it is clinically indicated and in the patient's best interests, and it's best practice for the patient, then we would prescribe and order them to patients… I am also thinking about the cost benefits of the length of stay… I think the benefits outweigh the disadvantages’. (OT001)
Motivation – Reflective	Goals (Action planning)	Audit and feedback mechanism in a multidisciplinary meeting monitors and improves pressure injury care—Facilitator	‘I think an increasing discussion about pressure injuries on the ward would be good… Like a safety huddle to chat about pressure injury incidence and cases at a multidisciplinary level… and things that don't happen on the ward that need to be worked on… I believe they generate those reports at the ward level. Still, we don't have as much access to those reports’. (OT004)
Opportunity – Physical	Environmental Context and Resources (Resources)	Ward‐based clinical champions facilitate pressure injury prevention—Facilitator	‘Our Grade 3 is quite present here and works full time. So I'm always able to go to her and ask some questions if I'm unsure… Having a clinical champion is a good idea. Having someone permanently there on the ward or a couple of people trained in that space’. (OT002)
Capability – Psychological	Knowledge (Knowledge)	Regular evidence updates and general pressure injury prevention education for all health disciplines—Facilitator	‘As the clinical workload increases, there's a little time lost to follow up on new procedures. So it's really good to have some time dedicated to learning about the most updated guidelines about pressure injuries… Some general understanding about the wounds and pressure care, the stages and malnutrition, all these basic understanding… It's important to have broad education across the team… so it's a real multidisciplinary approach’. (OT002)
Opportunity – Physical	Environmental Context and Resources (Resources)	Easily accessible evidence‐based checklists or flowcharts to prompt junior staff to assess patient pressure injury risk status comprehensively and make appropriate mattress decision—Facilitator	‘We have a flow chart in our policies and procedures, but there are a couple more factors that we need to consider as well, like cognition, continents… including this evidence in the flow chart to direct us and be a bit more specific would be more useful’. (OT002)

**FIGURE 1:**
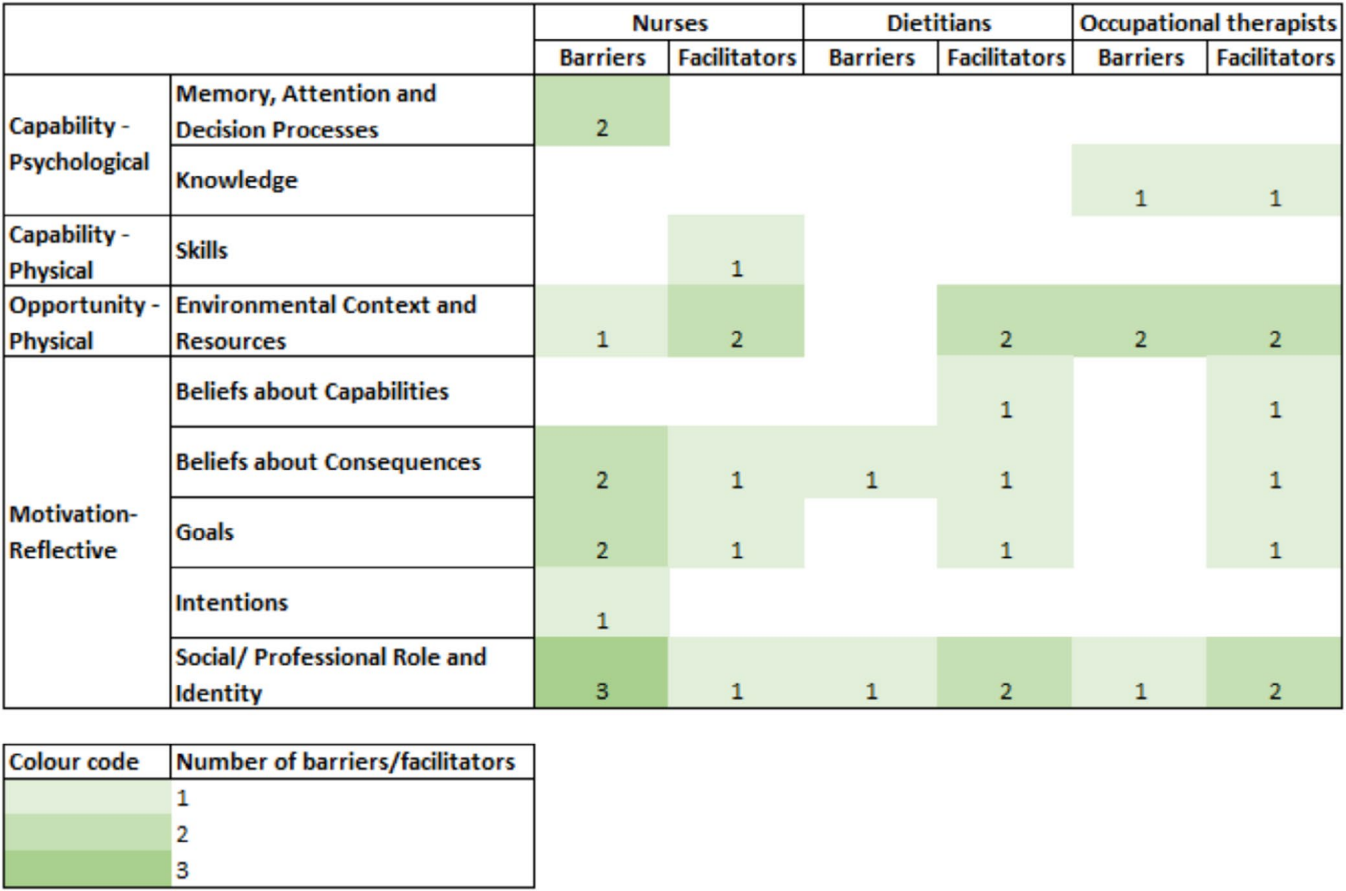
Number of barriers and facilitators identified in each capability, opportunity and motivation—behaviour model and Theoretical Domains Frameworks, stratified by health disciplines.

**FIGURE 2:**
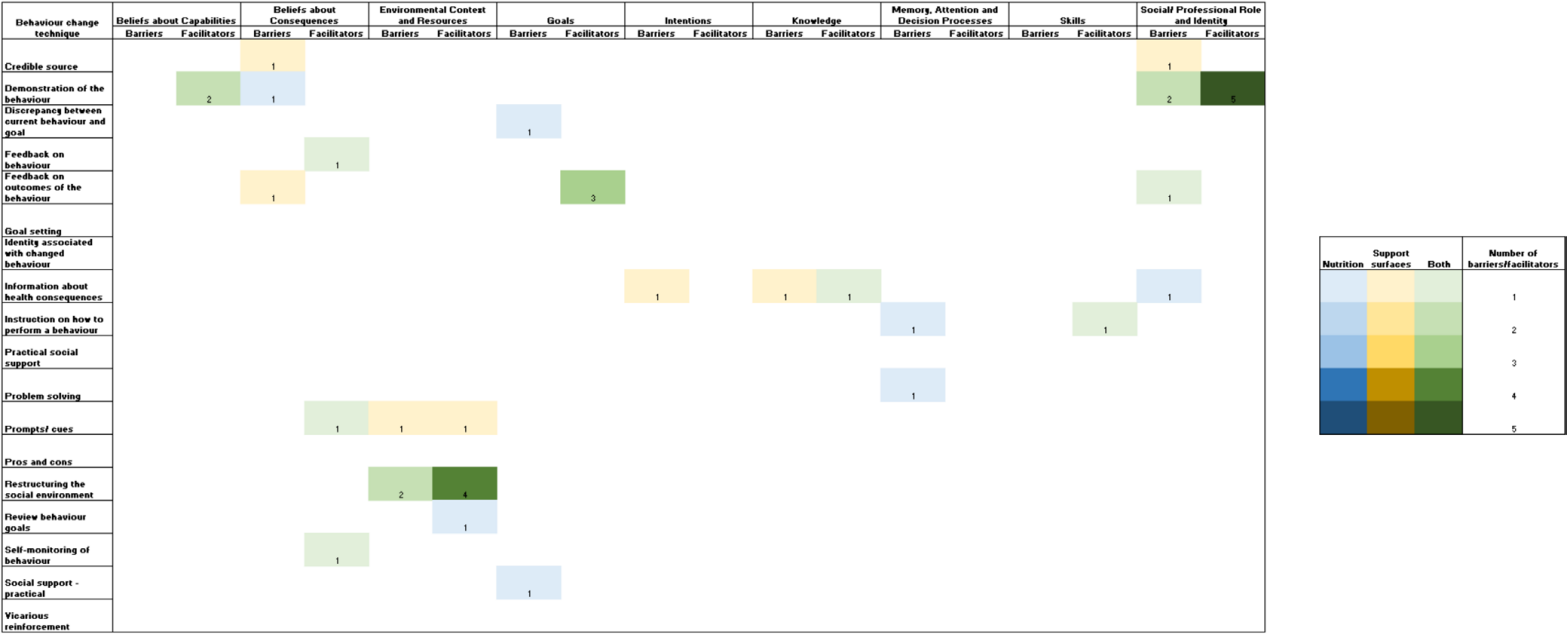
Number of barriers and facilitators mapped to behaviour change techniques from Theoretical Domains Framework.

Barriers and facilitators identified were then mapped from the nine Theoretical Domains Framework domains to 18 behaviour change techniques. Table S2 illustrates the behaviour change techniques allocation of barriers identified by the participants. Figure 1 provides an overview of the identified barriers and facilitators mapped to the Theoretical Domains Framework across health disciplines. Figure 2 shows the number of barriers and facilitators coded to specific behaviour change techniques allocated for each Theoretical Domains Framework domain. Barriers specific to nutrition and APAM were mapped to different behaviour change techniques, given the nature of different approaches in nutrition care implementation and APAM overuse de‐implementation.

The six themes are presented in detail below with illustrative quotes and the relevant Theoretical Domains Framework allocation.

### 4.1 Devaluing the Use of PI and Malnutrition Risk Assessment Tools

This theme described the incomplete or improper use of PI and malnutrition risk assessment tools administered by nurses because nurses devalued the use of these tools for facilitating clinical judgement on risk identification. They relied on clinical judgement based on their clinical knowledge, observations and experiences, which they believed could be used to comprehensively evaluate individual patients' risk without a structured risk assessment method (Memory, Attention and Decision Processes domain).

‘We pick up patients' health deterioration instead of filling out forms. Sometimes we don't use the form at all as a prompt… Clinical judgement is gained over years of experience’. (Nurse‐NFG002)

They prioritised vital sign‐related nursing tasks over risk assessment completion due to the difference in the magnitude of the emergency (Goals domain). They considered screening tools to be one type of paperwork on the to‐do list rather than a tool to guide clinical practice. They only used risk assessment tools as prompts to assess patients' risk when they were junior nurses (Memory, Attention and Decision Processes domain).

‘I remember when I first started, I did need prompting. Some things are easily missed, and you do need prompts… Particularly for junior colleagues, it [the assessment tool] is a good resource to return to if you feel like you've missed something for the day’. (Nurse‐NFG002)

Yet, dietitians mentioned missing referrals for patients at high risk of malnutrition because the nutritional screening tools were incomplete or completed improperly by nurses (Beliefs about Consequences domain). Additionally, some completed screening tools that indicated patients were at high risk of malnutrition failed to initiate a dietitian referral by nurses. If time permits, dietitians said they would screen all patients daily for malnutrition risk and send self‐referral if necessary to prevent missed referrals (Social/Professional Role and Identity domain).

‘Sometimes, with the Malnutrition Screening Tool, there are so many variations to how people use it. They might actually fill it in, and it's a risk, but do nothing with it… Sometimes, it's not filled in at all… It might be the patient's lost weight, but they [nurses] didn't ask the next question, “Did you try to lose weight?”… I might put in the referral myself’. (Dietitian‐D001)

Dietitians also expressed that nurses overlooked the role of nutrition care in PI prevention (Social/Professional Role and Identity domain). Nurses often considered nutrition support necessary for wound healing, not PI prevention. Therefore, patients were referred for PI management but not prevention. Similarly, nurses overlooked malnutrition risk assessment when evaluating patients' PI risks even though they knew malnutrition and PI were related (Memory, Attention and Decision Processes domain).

‘[Malnutrition screening] is separate from the pressure injury risk assessment. Malnutrition screening is different from fall risk and skin assessments… To be honest, we probably look at [malnutrition and pressure injury risk screening] separately. We don't think of them as hand in hand, which is probably not good. I think they are very relevant’. (Nurse‐NFG001)

### 4.2 Inadequately Integrate Nutrition Care Plan in Medical Treatment

This theme reflects the challenges nurses encountered in delivering nutrition care plans to patients at high risk of PI and malnutrition due to competing medical priorities such as diagnostic scans and medical therapies. Vital‐sign‐related medical treatments were viewed as more urgent than delivering nutrition care plans (Goals domain). Nurses described mealtime interruption as unavoidable when patients had to attend medical treatment during mealtime. During mealtime, nurses prioritised medical‐related nursing tasks, such as providing medications, over nutrition‐related nursing tasks, such as feeding assistance and food chart completion. They believed health assistants or nursing students could help with feeding assistance and food chart completion before food trays were removed by food service staff (Environmental Context and Resources domain).

‘We always get mealtime interrupted. There's always something… I think the nature of the hospital is that there are always people coming and going… Going for scans and having doctors seeing them [patients]… That's why they are here for… I think they are all necessary interruptions’. (Nurse‐NFG002)

To implement nutrition care plans as per nutrition‐related PI prevention guidelines, dietitians were more proactive in delivering individualised nutrition care plans by going to the wards during patients' mealtimes to observe, monitor and encourage dietary intake among patients at high risk of malnutrition (Social/Professional Role and Identity domain). They expressed the importance of raising awareness of the role nutrition plays in PI prevention among multidisciplinary team members, which was believed to help foster a desirable PI prevention work environment (Environmental Context and Resources domain).

‘We usually time our reviews at lunchtime, so we'll often be on the wards at a mealtime so we can be a physical support there and say, “This drink. Let's give it a try. Have a little bit more.”’ (Dietitian‐D002)

### 4.3 Overuse of Alternating Pressure Air Mattresses

This theme presents nurses' and junior occupational therapists' overreliance attitudes towards APAM use in PI prevention and lack of knowledge about the association between APAM use and repositioning frequency, resulting in the overuse of APAM as a PI prevention strategy to replace repositioning. All nurses and occupational therapists recognised and accepted that patients at low risk of PI were occasionally being put on an APAM. Senior occupational therapists mentioned it was due to the easy accessibility of APAMs and the lack of knowledge to support evidence‐based decision‐making on the relative benefits of using APAMs for patients at high risk of PIs among some nurses and junior occupational therapists (Knowledge domain). In addition, hospital guidelines prompted nurses to use APAM for patients at high risk of PI without considering patient independence (Environmental Context and Resources domain). Nurses did not acknowledge the significance of comprehensively evaluating the clinical need for APAM by considering patients' independence, recovery trajectory and discharge planning. When occupational therapists recommended downgrading mattresses as part of the discharge planning, they expressed that nurses were overcautious about putting patients at PI risk on a downgraded mattress because of nurses' perceived self‐accountability for PI prevention (Social/Professional Role and Identity domain).

‘I don't think we generally understand what mattress is used for which sort of patient… people are nervous that they don't want anything to go wrong on their watch. People often over‐prescribe and go for the strongest option rather than using their clinical reasoning because some nurses and junior occupational therapists don't have adequate clinical knowledge and clinical reasoning to look at other options. I think people are scared to do anything wrong… Because they [APAMs] are there, and they're ready to go, and it's really easy for us to do it, we give out so many’. (Occupational therapist‐OT003)

Similarly, regardless of the years of clinical nursing experience, nurses mentioned no issues accessing APAMs and believed APAM overuse in low PI risk patients was sometimes unavoidable when the previously discharged patients used APAM (Intentions domain). There was a time constraint in swapping back to standard beds before the new patient came. Some nurses viewed APAM as a one‐off quick‐fix strategy to replace continuous repositioning. Some nurses working in the subacute wards further suggested using APAMs as the standard hospital mattress because most patients in the subacute wards are at high risk of PI (Beliefs about Consequences domain).

‘I think the air mattress should be the standard in our geriatric ward… I think everyone should get an air mattress, so we don't need to order every time… Save time in changing beds… We think it is something that needs to be done more safely for patients to prevent pressure injuries’. (Nurse‐SFG002)

### 4.4 Nurses Coordinating Multidisciplinary Care

This theme described the significance of nurses' coordinating role in promoting interdisciplinary PI prevention care through effective interactions and communication within and between health disciplines. Nurses, dietitians and occupational therapists highlighted the importance of regular and reciprocal interaction between health disciplines to provide nutrition‐ and APAM‐related PI prevention care, which required an interdisciplinary approach in facilitating guideline uptake (Social/Professional Role and Identity domain). Nurses believed they play a key role in leading and coordinating the sharing of information between health disciplines to provide holistic PI prevention care (Social/Professional Role and Identity domain). Occupational therapists described more specifically the importance of role clarification in shared decision‐making between nurses and occupational therapists in pressure relief mattress prescription and evaluating mattress use according to patient recovery trajectory to enable appropriate mattress allocation throughout the patient's hospital stay (Social/Professional Role and Identity domain). Dietitians also highlighted the role of allied health assistants and food service staff in optimising nutrition care for PI prevention (Social/Professional Role and Identity domain).

‘A nurse is the one who makes referrals, implements the plan, and assists with information gathering… a holistic care approach between health professions’. (Nurse‐NFG004)

Yet, nurses were not confident in making mattress allocation decisions due to their lack of knowledge of pressure relief equipment expertise in PI prevention (Social/Professional Role and Identity domain). They relied on occupational therapists' expertise in making appropriate mattress allocation decisions. They expressed the need for having clearer professional boundaries regarding making or revising mattress allocation decisions according to patient recovery trajectory and mattress orders.

‘In the past, occupational therapists used to be much more involved than they are now… They will probably do the cushion, and they will do any home equipment… They used to do the air mattresses, and then they are less involved to the point where we nurses just order it because we know that if we don't do it, it'll never get done… It's part of the occupational therapist's job… That's part of their job title… It's better to have a clear role expectation for occupational therapists and nurses’. (Nurse‐SFG002)

Nurses also reported some dietitians and occupational therapists worked independently without sharing PI prevention care plans, which impeded multidisciplinary collaboration (Social/Professional Role and Identity domain). Some occupational therapists also highlighted interdisciplinary miscommunication with examples including a missed description of patients' PI risk status in referrals, missed occupational therapist referrals due to nurses' confusion about who would be automatically referred to occupational therapists in the system, and missed or delayed intradisciplinary handover of patients' PI prevention care plans in ward transfer (Environmental Context and Resources domain).

‘I think the ones that fall through the gaps are the patients whom we are not automatically involved with… It can be a bit confusing for the nursing staff to know who will automatically be picked up and who isn't… Pressure care is one of the clinical indicators that we could prioritise over certain other clinical indicators, but we are reliant on that information being communicated in our referral to aid that decision making to prioritise over other people’. (Occupational therapist‐OT004)

### 4.5 Facilitating Patient Participation in Their Care

This theme reveals that patient or carer participation in PI prevention was believed to promote guideline uptake. Yet, there were challenges in encouraging patient and carer (such as family members) involvement in PI prevention. Nurses, dietitians and occupational therapists valued patient or carer involvement in feeding assistance, food intake monitoring and mattress decision‐making to tailor to patients' preferences and needs (Environmental Conext and Resources domain). Overcoming language barriers was particularly valued with carer involvement. Participants believed that the earlier they involved patients and carers when appropriate, the quicker the PI and malnutrition risk would be identified and the more relevant and timely PI prevention strategies the patients would have.

‘They [patients] have to gain weight and become more nourished… Education resources to raise awareness of the importance of nutrition in pressure injury prevention would be useful… They have to help themselves and the desire to get better… You will need their cooperation’. (Nurse‐NFG002)

Both dietitians and occupational therapists felt capable of involving patients and carers in nutrition and mattress decision‐making, respectively (Beliefs about Capabilities domain). Given dietitians' and occupational therapists' expertise in nutrition and mattresses, nurses preferred to delegate patient engagement and involvement in these PI prevention care to dietitians and occupational therapists (Social/Professional Role and Identity domain). Nurses found involving patients in mattress decision‐making particularly challenging due to the perceived consequences of patients refusing to APAM despite clinical needs and their lack of knowledge about the benefits and drawbacks of different mattress options (Beliefs about Consequences domain).

‘The education is just an idea to explain to family members when they come to ask us questions… we get education about the wound products and how to dress that patient… But I have never been exposed to and to educate mattresses, so it is very hard to explain to patients why we're using this particular one [APAM] but not an alternative one’. (Nurse‐SFG002)

### 4.6 Beliefs About the Value of PI Prevention Guidelines

This theme presented shared views between nurses, dietitians and occupational therapists on the perceived significance of accessible PI prevention guidelines in providing evidence‐based practice, improving patient outcomes in PI prevention and reducing unnecessary hospital costs and resource use that contribute to PIs. They believed these benefits outweighed the increased workload as drawbacks of following guidelines. Nurses and occupational therapists thought no additional workload was required to implement clinical guidelines as their current practice did not deviate from them (Beliefs about Consequences domain). Yet, dietitians anticipated that by implementing PI prevention guidelines, more patients would be screened for nutritional assessments and developing meal plans (Beliefs about Consequences domain).

‘Pressure injury prevention is just a routine thing. I don't think following guidelines would create an additional workload for us because we are following guidelines anyway’. (Nurse‐SFG001)

‘If we implemented these guidelines, the biggest change would be the nutrition time. I wouldn't anticipate that it would be a huge amount of extra time for nursing staff to complete a screening tool. Potentially for the dietitians, it might be a barrier to the time to conduct more assessments and implement more care plans… which is needed’. (Dietitian‐D002)

Both nurses, dietitians and occupational therapists believed that audit and feedback to be used in a multidisciplinary meeting (Goals domain), and ward‐based clinical champions might facilitate the implementation of PI prevention guidelines, fostering PI prevention culture and improving PI prevention care (Environmental Context and Resources domain). Dietitians and occupational therapist clinical champions were recommended to ‘demonstrate the desirable behaviour’ as a behaviour change technique to role‐model PI prevention practices. Introducing ward‐based nursing clinical champions was suggested to ‘restructure the social environment’ as a behaviour change technique to facilitate the practice change.

Due to high nursing staff turnover, nurses particularly highlighted the importance of ongoing PI prevention education and training to provide bedside nurses with updated knowledge and skills in PI prevention (Skills domain). Occupational therapists also described the significance of general PI prevention education for all health disciplines to foster a PI prevention culture, regular evidence updates on mattresses to enhance evidence‐based care (Knowledge domain) and easily accessible evidence‐based checklists or flowcharts to prompt evidence‐informed mattress allocation decisions (Environmental Context and Resources domain).

‘I think something like a checklist would be useful, or like a flow chart, something that's easy to refer to. Because sometimes, when you're really busy clinically, you don't have time to go and read through all of the journal articles’. (Occupational therapist‐OT001)

## 5 Discussion

To our knowledge, this is the first qualitative study that explored hospital nurses', dietitians' and occupational therapists' views on barriers and facilitators to implementing PI prevention guidelines related to nutrition assessment and treatment and de‐implementing APAM overprescription. Clinicians' attitudinal barriers to implementing these nutrition‐ and APAM‐related guideline recommendations are key drivers for these problems. They valued a nurse‐led interdisciplinary approach that promotes patient and carer involvement to improve PI prevention care. Unlike allied health views, nurses mentioned difficulties integrating nutrition support in medical care and patient involvement in the decision‐making around mattress selection. Barriers and facilitators derived from the findings were mapped to Theoretical Domains Framework and behaviour change techniques for future intervention development.

The key barrier in this study related to underusing structured PI and malnutrition screening tools to complement nurses' clinical judgement, as nursing staff believed their clinical judgement alone was more comprehensive for assessing patients' PI and malnutrition risk. Yet, it is unclear using nursing clinical judgement better predicts patient risk status than using structured risk assessment tools (Lovegrove, Ven, Miles, and Fulbrook, 2021). Despite this, it has been shown that nurses are more prone to overlook nutrition‐related problems related to skin vulnerability when making clinical judgements about PI risks (Van Den Berg et al. 2023). Only 20% of hospital inpatients were screened for malnutrition risk, and preventative strategies were only targeted to inpatients with a PI instead of inpatients at PI risk (Tervo‐Heikkinen et al. 2023). Of concern is that up to 65% of hospital inpatients experience a nutritional decline in their hospital stay (Cass and Charlton 2022). Early identification of malnutrition risk and timely nutritional intervention are critical to preventing hospital‐acquired malnutrition and improving patient outcomes (Cass and Charlton 2022). Yet, nurses complete the malnutrition screening tools improperly as they are not trained to use them (Eglseer et al. 2018). The improper use of malnutrition screening tools might contribute to false‐positive dietitian referrals, which increase unnecessary workload (Phillips and Zechariah 2017). Regular education on malnutrition risk factors and ongoing nurse training on completing malnutrition screening tools may increase their awareness of malnourishment when considering patient PI risks and enhance the accuracy of identifying patients with malnutrition risk (Marshall et al. 2018).

Regarding APAM overuse, the key drivers of the APAM overprescription to patients at low PI risk were that nurses used APAM as a one‐off quick‐fix strategy to replace regular repositioning in clinical settings with competing priorities. They were over‐reliant on APAM to prevent PI and hence overcautious about PI risk related to downgrading APAM. These findings provide new insights into existing literature about what potential low‐value PI care can result if the organisational‐level barrier of pressure‐relieving mattress shortage is addressed, leaving behind the individual‐level barrier related to nurses' lack of updated knowledge about pressure relief equipment use (Wan et al. 2023). According to the best available evidence on APAM use, patients who can self‐reposition should not be put on an APAM (Huang et al. 2023) due to its negative impact on patients' independent movement and comfort (Nixon et al. 2019). Replacing regular repositioning as a fundamental PI prevention strategy (Alshahrani et al. 2021) with APAM overuse impedes patient safety. Mattress decision‐making should be informed by patient preference, rehabilitation needs and other modifiable risk factors such as immobility and nutritional deficits (Nixon et al. 2019). Patient and carer involvement in mattress shared decision‐making is hence critical. Given occupational therapists' expertise in pressure relief equipment, occupational therapists might be better positioned to involve patients and carers in the shared decision‐making process. Our participants also reflected that a clinical decision aid in supporting evidence‐informed decision‐making on mattress use might be useful to junior nurses and occupational therapists. As the most recent mattress decision tool was published in 2005 (Wall et al. 2005), future research is needed to update an evidence‐based mattress decision flowchart.

Another key finding of this study was the advocacy for nurse‐led interdisciplinary collaboration and patient and carer involvement in PI prevention care as perceived facilitators to improve guideline uptake. Implementing nutrition‐ and APAM‐related PI prevention guidelines requires an effective interprofessional collaboration model of care in an acute hospital setting. However, the evidence on strategies to support interdisciplinary team‐based PI practices is scarce (Suva et al. 2018). Similarly, for patient participation, engaging patients in shared clinical decision‐making using patient‐targeted educational materials or shared decision‐making tools is effective in de‐implementing low‐value practices and improving high‐quality, sustainable healthcare (Sypes et al. 2020). Yet, these strategies were only evaluated in primary care (Sypes et al. 2020). Successful patient engagement is context‐specific and depends on the clinician's motivation and self‐efficacy of patient involvement, as well as the availability of shared decision support resources (Scott et al. 2021). Given the multiple barriers identified in previous literature on improving interdisciplinary care and patient participation in hospitals (Wan et al. 2023), identifying priority barriers using a stakeholder partnership approach in future research will help guide the intervention development (Gillespie et al. 2020).

### 5.1 Limitations

This study has several limitations. First, it was conducted in a single hospital, so the findings may not be generalisable to other hospitals. Yet, we sampled clinicians from different health disciplines with a broad range of clinical experiences and roles who worked in the wards that admitted patients at high risk of PI. This provided an in‐depth and breadth of understanding of current multidisciplinary PI prevention practices that are transferable to other hospital settings. Second, this study only included nurses, dietitians and occupational therapists as key healthcare providers of nutrition‐ and APAM‐related PI prevention practices. Physicians' and physiotherapists' views on general PI prevention practices and multidisciplinary care were not captured. Due to the explorative nature of the study design, the interview guide was not piloted before data collection. Yet, the interview guide was developed by researchers with nursing, dietetics and occupational therapist backgrounds to enhance clinical relevancy in exploring current PI prevention practices.

## 6 Conclusions and Recommendations for Further Research

Given the context‐specific nature of nutrition‐ and APAM‐related PI prevention problems identified in this study, the implementation strategies allocated to address barriers related to nutrition‐ and APAM‐related PI prevention are different. A theory‐driven, evidence‐informed intervention specific to nutrition and mattresses is required. As a nurse‐led interdisciplinary approach that promotes patient and carer participation is considered imperative for nutrition‐ and APAM‐related PI prevention, strategies to improve interdisciplinary interaction and communication, and patient and carer involvement in decision‐making may need to be applied to nutrition‐ and APAM intervention development. The feasibility and effectiveness of the multifaceted interventions to improve nutrition‐ and APAM‐related PI prevention based on the findings of this study will be evaluated in future pilot trials.

## 7 Implications for Clinical Practice

To improve nutrition‐related PI prevention care, when assessing PI risk using clinical judgement, nurses need to be reminded to consider undernutrition‐related skin vulnerability. Nurses' education on predictors of malnutrition in hospital inpatients and training on completing evidence‐informed risk assessment tools might help alleviate misconceptions of malnutrition among nursing staff and improve malnutrition screening accuracy. Regarding improving APAM‐related PI prevention care, raising the awareness of the need to reposition regularly regardless of the choice of mattresses and adjusting repositioning frequency when a pressure relief mattress is used are critical to de‐implement potential APAM overuse.

## Author Contributions

C.S.W., B.G., G.T. and E.M. contributed to the conception and design of the research. C.S.W. and M.M. were responsible for conducting the research, data management and analysis. C.S.W. was responsible for writing the initial draft of the manuscript. B.G., G.T. and E.M. also contributed to the interpretation and presentation of the findings. All authors critically revised the manuscript, agreed to be fully accountable for ensuring the integrity and accuracy of the work, and read and approved the final manuscript.

## Conflicts of Interest

The authors declare no conflicts of interest.

## Peer Review

The peer review history for this article is available at https://www.webofscience.com/api/gateway/wos/peer‐review/10.1111/jan.16820.

## Supporting information


**Table S1:** Overview of bedside nurses', dietitians' and occupational therapists' views on barriers and facilitators to implementing nutrition‐ and alternating pressure air mattress‐related pressure injury prevention guidelines.
**Table S2:** Subthemes presented as barriers and facilitators identified mapping to the intervention functions based on the behaviour change wheel (BCW) and behaviour change technique (BCT).

## Data Availability

The data that support the findings of this study are available on request from the corresponding author and participants. The data are not publicly available due to privacy or ethical restrictions.
