# Supplementary material for: Barriers and Facilitators to Implementing Pressure Injury Guidelines for Nutrition Assessment and Alternating Pressure Air Mattress Allocation: A Qualitative Study
Source: J Adv Nurs. 2025 Feb 12;81(10):6767–88. doi: 10.1111/jan.16820 (PMC12460943; doi:10.1111/jan.16820)
Supplement: Supplementary file 1 — Table S1: Overview of bedside nurses', dietitians' and occupational therapists' views on barriers and facilitators to implementing nutrition‐ and alternating pressure air mattress‐related pressure injury prevention guidelines. Table S2: Subthemes presented as barriers and facilitators identified mapping to the intervention functions based on the behaviour change wheel (BCW) and behaviour change technique (BCT). [file JAN-81-6767-s001.docx]

Supplementary Table 1: Overview of bedside nurses’, dietitians’ and occupational therapists’ views on barriers and facilitators to implementing nutrition- and alternating pressure air mattress-related pressure injury prevention guidelines

| Themes | Bedside nurses’ views | Dietitians’ views | Occupational therapists’ views |
| --- | --- | --- | --- |
| Theme 1: Devaluing of the use of pressure injury and malnutrition risk assessment tools | *Barriers:*  1. Prioritise vital sign-related nursing tasks over risk assessment completion  2. Only use risk assessment tools as prompts when nurses feel they are unable to make a clinical judgement to decide if referral to allied health is needed  3. Do not consider malnutrition risk when assessing pressure injury risk because separate assessment tools are used | *Barriers:*  1. Missed referrals for patients at high risk of malnutrition because Malnutrition Screening Tools are incomplete or completed improperly by nurses  2. Nurses overlook the role of nutrition care in pressure injury prevention | Not applicable |
| Theme 2:  Inadequately integrate nutrition care plan in medical treatment | *Barriers:*  1. Prioritise medical intervention over patient mealtime  2. Incomplete food charts due to other nursing workload priorities and hence food intake monitoring is suboptimal | *Facilitators:*  1. Hospitals need to raise awareness of nutrition in pressure injury prevention among the multidisciplinary team  2. Monitor and encourage dietary intake of patients who are at risk of malnutrition during mealtimes | Not applicable |
| Theme 3: Overuse of alternating pressure air mattresses | *Barriers:*  1. Alternating pressure air mattresses overuse is safer for patients than underuse to prevent pressure injuries  2. Some nurses prefer alternating pressure air mattresses to be the standard hospital mattress because most patients are at risk of pressure injuries | Not applicable | *Barriers:*  1. Over-ordering of alternating pressure air mattresses by nurses due to easy accessibility and lack of knowledge of the benefits and drawbacks of all mattress options among nurses and junior occupational therapists  2. Hospital guidelines direct nurses to use alternating pressure air mattresses in patients at high risk of pressure injuries regardless of patient independence  3. Nurses concern about bearing risks associated with downgrading mattresses |
| Theme 4: Nurses coordinating multidisciplinary care | *Facilitators:*  1. Importance of nurses’ coordinating role in providing regular and reciprocal interactions between the multidisciplinary team in pressure injury prevention *Barriers:*  2. Lack of expertise to be solely responsible for making decisions regarding mattress allocation  3. Some dietitians and occupational therapists work independently with little multidisciplinary communication | *Facilitators:*  1. Collaborative teamwork between nurses, allied health and food service staff is important for optimising nutrition care for pressure injury prevention | *Facilitators:*  1. Clarification of the role of the multidisciplinary team in relation to prescription and monitoring the use of support surfaces  2. Input from multidisciplinary team comprising nurses and occupational therapists to enable shared decision-making in prescribing appropriate mattresses  *Barriers:*  3. Suboptimal multidisciplinary communication and intradisciplinary handover of patient’s pressure injury prevention plan |
| Theme 5:  Facilitating patient participation in their care | *Facilitators:*  1. Encourage family involvement in feeding patients and providing communication assistance between the patient and nurses  *Barriers:*  2. Not confident in involving patients in the nutrition care plans and mattresses allocation decision-making due to nurses’ lack of expertise in nutrition and mattress  3. Challenging to involve patients in mattress decision-making because patients dislike alternating pressure air mattresses regardless of pressure injury risk | *Facilitators:*  1. Value patient or family involvement in food chart completion | *Facilitators:*  1. Encourage shared decision-making in allocating appropriate mattresses by involving patients and/or carers early |
| Theme 6: Beliefs about the value of pressure injury prevention guidelines | *Facilitators:*  1. Following guidelines helps deliver positive outcomes without increasing workload  2. Audit and feedback mechanism in a multidisciplinary meeting improves pressure injury care  3. Ward-based clinical champions lead and coordinate multidisciplinary pressure injury prevention  4. Ongoing pressure injury prevention education and training for bedside nurses | *Facilitators:*  1. Following pressure injury prevention guidelines is essential despite the increased workload in conducting nutritional assessments  2. Audit and feedback mechanism in a multidisciplinary meeting improves pressure injury care  3. Pressure injury prevention clinical champion in each ward monitors the appropriateness of dietitian referral | *Facilitators:*  1. Prefer following guidelines because the benefits of following guidelines outweigh the drawbacks  2. Audit and feedback mechanism in a multidisciplinary meeting monitors and improves pressure injury care  3. Ward-based clinical champions facilitate pressure injury prevention  4. Regular evidence updates and general pressure injury prevention education for all health disciplines  5. Easily accessible evidence-based checklists or flowcharts to prompt junior staff to assess patient pressure injury risk status comprehensively and make appropriate mattress decision |

Supplementary Table 2: Subthemes presented as barriers and facilitators identified mapping to the intervention functions based on the Behaviour Change Wheel (BCW) and behaviour change technique (BCT)

1. ***Nurses***

| **Subtheme presented as a barrier** | **Intervention functions based on BCW** | **Intervention strategies based on BCT (Details of the intervention)** |
| --- | --- | --- |
| **Theme 1: Devaluing of the use of pressure injury and malnutrition risk assessment tools** | | |
| Prioritise vital sign-related nursing tasks over risk assessment completion - Barrier | Persuasion | Discrepancy between current behaviour and goal  (Point out that both monitoring clinical vital signs and skin integrity are nurse’s responsibilities in a multidisciplinary setting and should be prioritised as part of the routine nursing practice) |
| Only use risk assessment tools as prompts when nurses feel they are unable to make a clinical judgement to decide if referral to allied health is needed - Barrier | Enablement | Problem solving  (Prompt evidence-informed nursing clinical judgement on dietitian referral using an evidence-based malnutrition screening flowchart to help identify patients at high risk of malnutrition or who are malnourished) |
| Do not consider malnutrition risk when assessing pressure injury risk because separate assessment tools are used - Barrier | Training | Instruction on how to perform a behaviour  (Imparting skills of how to use the evidence-based malnutrition screening flowchart to write appropriate reasons for dietitian referrals to facilitate dietitians prioritising their clinical workload to patients at high risk of pressure injuries) |
| **Theme 2: Inadequately integrate nutrition care plan in medical treatment** | | |
| Prioritise medical intervention over patient mealtime - Barrier | Enablement | Social support - practical  (Seek additional support from dietitians to revise nutrition care plan for patients who constantly require medical intervention) |
| Incomplete food charts due to other nursing workload priorities and hence food intake monitoring is suboptimal - Barrier | Enablement | Restructuring the social environment  (Dietitians to evaluate the individual need for completing food charts and communicate with the bedside nurses regularly so bedside nurses can prioritise their workload away from completing unnecessary food charts) |
| **Theme 3: Overuse of alternating pressure air mattresses** | | |
| Alternating pressure air mattresses overuse is safer for patients than underuse to prevent pressure injuries - Barrier | Education | Information about health consequences  (Face-to-face group education to bedside nurses on the potential patient harm of the overuse of alternating pressure air mattress in addition to increased hospital cost) |
| Some nurses prefer alternating pressure air mattresses to be the standard hospital mattress because most patients are at risk of pressure injuries - Barrier | Persuasion | Feedback on outcomes of the behaviour  (In the face-to-face group education with bedside nurses, highlighting the importance of appropriate mattress allocation to minimise patient harm related to alternating pressure air mattress overuse) |
| **Theme 4: Nurses coordinating multidisciplinary care** | | |
| Importance of nurses’ coordinating role in providing regular and reciprocal interactions between the multidisciplinary team in pressure injury prevention - Facilitator | Modelling | Demonstration of the behaviour  (Ward-based nursing clinical champions demonstrate optimal interdisciplinary pressure care and assist with interdisciplinary communication) |
| Lack of expertise to be solely responsible for making decisions regarding mattress allocation - Barrier | Modelling | Demonstration of the behaviour  (Ward-based nursing clinical champions demonstrate how to include occupational therapists in shared decision-making about appropriate mattress allocation) |
| Some dietitians and occupational therapists work independently with little multidisciplinary communication - Barrier | Persuasion | Feedback on outcomes of the behaviour  (Emphasise the significance of the dietitians’ and occupational therapists’ roles in pressure injury prevention and suggest working with ward-based nursing clinical champions to improve interdisciplinary pressure care) |
| **Theme 5: Facilitating patient participation in their care** | | |
| Encourage family involvement in feeding patients and providing communication assistance between the patient and nurses - Facilitator | Enablement | Restructuring the social environment  (Advise bedside nurses to involve family members in pressure injury care via phone or in person if possible) |
| Not confident in involving patients in the nutrition care plans and mattresses allocation decision-making due to nurses’ lack of expertise in nutrition and mattress - Barrier | Modelling | Demonstration of the behaviour  (Ward-based nursing clinical champions demonstrate how to communicate and advocate patients' preferences on nutrition and mattresses to dietitians and occupational therapists) |
| Challenging to involve patients in mattress decision-making because patients dislike alternating pressure air mattresses regardless of pressure injury risk - Barrier | Persuasion | Credible source  (Present audio recordings from the health consumers focus group to clarify their willingness to use alternating pressure air mattresses if they know they are at high risk of pressure injuries) |
| **Theme 6: Beliefs about value of pressure injury prevention guidelines** | | |
| Following guidelines helps deliver positive outcomes without increasing workload - Facilitator | Education | Prompts/cues  (Emphasise the importance of optimal nutrition care and appropriate mattress allocation in pressure injury prevention as stated in clinical guidelines. Suggest bedside nurses use malnutrition screening and mattress flowcharts to prompt evidence-based clinical judgement) |
| Audit and feedback mechanism in a multidisciplinary meeting improves pressure injury care - Facilitator | Persuasion | Feedback on outcomes of the behaviour  (Inform nurse managers, dietitians, occupational therapists and ward-based nursing clinical champions about malnutrition and pressure injury incidence, alternating pressure air mattress use and patient satisfaction monthly and provide opportunities to discuss strategies to improve pressure injury care) |
| Ward-based clinical champions lead and coordinate multidisciplinary pressure injury prevention - Facilitator | Environmental restructuring | Restructuring the social environment  (Nominate ward-based nursing clinical champions to lead practice change and coordinate interdisciplinary communication) |
| Ongoing pressure injury prevention education and training for bedside nurses - Facilitator | Training | Instruction on how to perform a behaviour  (Regular bedside nurse training on updated evidence-based strategies to prevent pressure injuries including malnutrition screening and appropriate mattress allocation) |

BCT: Behaviour Change Technique; BCW: Behaviour Change Wheel

1. ***Dietitians***

| **Subtheme presented as a barrier** | **Intervention functions based on BCW** | **Intervention strategies based on BCT (Details of the intervention)** |
| --- | --- | --- |
| **Theme: Devaluing of the use of pressure injury and malnutrition risk assessment tools** | | |
| Missed referrals for patients at high risk of malnutrition because MSTs are incomplete or completed improperly by nurses - Barrier | Modelling | Demonstration of the behaviour  (Dietitians demonstrate to bedside nurses how to do malnutrition screening) |
| Nurses overlook the role of nutrition care in pressure injury prevention - Barrier | Education | Information about health consequences  (Beside nurse education on the association between malnutrition and pressure injury incidence) |
| **Theme: Inadequately integrate nutrition care plan in medical treatment** | | |
| Hospitals need to raise awareness of nutrition in pressure injury prevention among the multidisciplinary team - Facilitator | Environmental restructuring | Restructuring the social environment  (Ward-based nursing clinical champions facilitate communication between bedside nurses and dietitians to provide nutrition support to patients at high risk of pressure injuries) |
| Monitor and encourage dietary intake of patients who are at risk of malnutrition during mealtimes - Facilitator | Modelling | Demonstration of the behaviour  (Senior dietitians demonstrate to junior dietitians how to monitor and encourage the dietary intake of patients during patient mealtimes) |
| **Theme: Nurses coordinating multidisciplinary care** | | |
| Collaborative teamwork between nurses, allied health and food service staff is important for optimising nutrition care for pressure injury prevention - Facilitator | Modelling | Demonstration of the behaviour  (Senior dietitians demonstrate to junior dietitians how to collaborate with other health professions to implement individualised nutrition care plans) |
| **Theme: Facilitating patient participation in their care** | | |
| Value patient or family involvement in food chart completion - Facilitator | Modelling | Demonstration of the behaviour  (Senior dietitians demonstrate to junior dietitians how to involve patients and/or family members in food chart completion and dietary intake monitoring) |
| **Theme: Beliefs about value of pressure injury prevention guidelines** | | |
| Following pressure injury prevention guidelines is essential despite the increased workload in conducting nutritional assessments - Facilitator | Persuasion | Feedback on behaviour  (Inform dietitians that in order to follow guidelines, they only need to prioritise patients at high risk of pressure injuries to be seen) |
| Audit and feedback mechanism in a multidisciplinary meeting improves pressure injury care - Facilitator | Persuasion | Feedback on outcomes of the behaviour  (Inform nurse managers, dietitians, occupational therapists and ward-based nursing clinical champions about pressure injury incidence, alternative pressure air mattress use and patient satisfaction monthly and provide opportunities to discuss strategies to improve pressure injury care) |
| Pressure injury prevention clinical champion in each ward monitors the appropriateness of dietitian referral - Facilitator | Enablement | Review behaviour goals  (Ward-based nursing clinical champions talk to bedside nurses monthly about how well they use the malnutrition screening flowchart to guide clinical judgement and provide support if needed) |

BCT: Behaviour Change Technique; BCW: Behaviour Change Wheel; MST: Malnutrition Screening Tool

1. ***Occupational therapists***

| **Subtheme presented as a barrier** | **Intervention strategies based on BCW** | **Intervention strategies based on BCT (Details of the intervention)** |
| --- | --- | --- |
| **Theme: Overuse of alternating pressure air mattresses** | | |
| Over-ordering of alternating pressure air mattresses by nurses due to easy accessibility and lack of knowledge of the benefits and drawbacks of all mattress options among nurses and junior occupational therapists - Barrier | Education | Information about health consequences  (Education to junior bedside nurses and occupational therapists on the benefits and drawbacks of alternating pressure air mattress use to empower them with making evidence-based clinical judgement) |
| Hospital guidelines direct nurses to use alternating pressure air mattresses in patients at high risk of pressure injuries regardless of patient independence - Barrier | Environmental restructuring | Prompts/ cues  (Bedside nurses use mattress flow charts to prompt evidence-based decision making on mattress allocation) |
| Nurses concern about bearing risks associated with downgrading mattresses - Barrier | Persuasion | Credible source  (Ward-based nursing clinical champions encourage bedside nurses who are concerned about downgrading mattresses to discuss it with occupational therapists to come up with a mutually agreeable plan for mattress use) |
| **Theme: Nurses coordinating multidisciplinary care** | | |
| Clarification of the role of the multidisciplinary team in relation to prescription and monitoring the use of support surfaces - Facilitator | Modelling | Demonstration of the behaviour  (Ward-based nursing clinical champions demonstrate professional boundaries between bedside nurses and occupational therapists in prescribing and monitoring appropriate mattress allocation) |
| Input from multidisciplinary team comprising nurses and occupational therapists to enable shared decision-making in prescribing appropriate mattresses - Facilitator | Modelling | Demonstration of the behaviour  (Ward-based nursing clinical champions demonstrate how to work with occupational therapists to enable shared decision-making in appropriate mattress allocation) |
| Suboptimal multidisciplinary communication and intradisciplinary handover of patient’s pressure injury prevention plan - Barrier | Environmental restructuring | Restructuring the social environment  (Ward-based nursing clinical champions facilitate communication between bedside nurses, dietitians and occupational therapists by providing an updated overview of pressure injury prevention plans for patients at high risk of pressure injuries in a weekly multidisciplinary team meeting) |
| **Theme: Facilitating patient participation in their care** | | |
| Encourage shared decision-making in allocating appropriate mattresses by involving patients and/or carers early - Facilitator | Modelling | Demonstration of the behaviour  (Senior occupational therapists demonstrate to junior staff how to involve patients and/or family members in mattress decision-making) |
| **Theme: Beliefs about value of pressure injury prevention guidelines** | | |
| Prefer following guidelines because the benefits of following guidelines outweigh the drawbacks - Facilitator | Education | Self-monitoring of behaviour  (Relate the evidence-based mattress flowchart to clinical guidelines and ask occupational therapists to record how often they use the mattress flowchart to facilitate clinical judgment on alternative pressure air mattress use) |
| Audit and feedback mechanism in a multidisciplinary meeting monitors and improves pressure injury care - Facilitator | Persuasion | Feedback on outcomes of the behaviour  (From bedside nurses' feedback on problems with interdisciplinary communication, group discussion in a weekly multidisciplinary team meeting with nurse managers, dietitians, occupational therapists and ward-based nursing clinical champions about strategies to improve interdisciplinary collaboration in providing pressure injury prevention care) |
| Ward-based clinical champions facilitate pressure injury prevention - Facilitator | Environmental restructuring | Restructuring the social environment  (Nominate ward-based nursing clinical champions to coordinate interdisciplinary communication between bedside nurses, physiotherapists and occupational therapists) |
| Regular evidence updates and general pressure injury prevention education for all health disciplines - Facilitator | Education | Information about health consequences  (Ongoing education on updated knowledge about strategies to prevent pressure injuries among bedside nurses, dietitians and occupational therapists, and emphasise the importance of interprofessional collaboration to prevent pressure injuries) |
| Easily accessible evidence-based checklists or flowcharts to prompt junior staff to assess patient pressure injury risk status comprehensively and make appropriate mattress decision - Facilitator | Environmental restructuring | Prompts/cues  (Evidence-based mattress flowchart is accessible to occupational therapists to facilitate clinical judgment on appropriate mattress use) |

BCT: Behaviour Change Technique; BCW: Behaviour Change Wheel
